# Multi-Sector Collaboration for Improving Home-Based Nutrition Supports: Process Findings from a Clinical Trial

**DOI:** 10.1093/geroni/igab046.2869

**Published:** 2021-12-17

**Authors:** David Wihry, Jennifer Jain, Jennifer Crittenden

**Affiliations:** 1 University of Maine, Bangor, Maine, United States; 2 University of Maine, University of Maine, Maine, United States

## Abstract

In-home supports and proper nutrition are critical to post-acute recovery and long-term health management for adults 60 and older. At the same time, such supports are often difficult to deploy in rural settings. To address these challenges, a unique multi-sector consortia was formed between a local Area Agency on Aging, a healthcare system, a health technology company, and a university to conduct a clinical trial of a novel in-home health technology program coupled with customized chronic care nutrition support. Early stage clinical trial development required coordination across health and community-based organizations to develop a pathway for older adults to access the in-home project supports. At the conclusion of the year one, six project partners were interviewed using a semi-structured interview protocol examining the strengths of early project design and challenges inherent in the early phases of a community-based clinical trial. Thematic analysis uncovered six themes instructive in formulating efficacious clinical trial methodologies: 1) Logistical challenges related to the pandemic, including reduced patient numbers and the curtailing of in-hospital recruitment; 2) Partner collaboration as essential to designing preferred project modifications; 3) The challenge of converting project referrals into project enrollees; 4) A new appreciation among community partners regarding institutional review board requirements; 5) Recommendations for addressing emerging staffing challenges; and 6) The overriding importance of engaging older adults in their own care and health promotion post-discharge. Results will inform construction of a replicable model for establishing novel research partnerships that span healthcare, social services, the business sector, and higher education.

